# Fear of progression after cancer recurrence: a mixed methods study

**DOI:** 10.3389/fpsyg.2024.1479540

**Published:** 2024-09-25

**Authors:** Ross James Stewart, Gerald Michael Humphris, Jayne Donaldson, Susanne Cruickshank

**Affiliations:** ^1^Faculty of Health Sciences and Sport, University of Stirling, Stirling, United Kingdom; ^2^School of Medicine, University of St. Andrews, St. Andrews, United Kingdom; ^3^The Royal Marsden NHS Foundation Trust, London, United Kingdom

**Keywords:** cancer recurrence, fear of progression, fear of recurrence, quality of life, oncology

## Abstract

**Background:**

The recurrence of cancer will significantly impact an individual’s quality of life (QoL) as they adjust to living with a condition that is often incurable. Patients remain at risk of further progression following recurrence, but fear of cancer progression (FOP) at this time is not commonly examined. Importantly, these fears are known to reach levels in which there are consequences for QoL.

**Methods:**

This study sought to explore levels of FOP, health-related QoL, anxiety, and depression in patients after a recurrence of their cancer in a longitudinal manner. With the study taking place throughout the COVID-19 pandemic, an assessment of fears related to cancer and the pandemic was included. A sequential mixed method approach was employed for complementarity and expansion purposes. A questionnaire was administered to 44 participants on three different occasions one month apart. A sub-sample of 10 participants then took part in semi-structured interviews.

**Findings:**

FOP was present at moderate levels in patients with a cancer recurrence, with over a third of the sample reaching levels considered dysfunctional. Levels of fear were stable over three months and were not predicted by select demographic or clinical factors. On average, depression was low, but anxiety reached mild levels. Challenges to health-related QoL were evident. Low levels of concern about COVID-19 in relation to cancer were reported. Integrated findings provided more nuanced answers to the research questions, including more specific worries about cancer progression.

**Implications:**

Findings support the development of psychosocial interventions to manage FOP, and future recommendations are provided. Identifying the presence of fears not commonly screened for after cancer recurrence adds to the existing knowledge in this area. Through acknowledging and attending to the psychosocial impact of FOP, healthcare professionals can provide tailored support to enhance the well-being of those with a recurrence of their cancer.

## 1 Introduction

Despite improvements in cancer survival, patients remain at risk of recurrence and progression, particularly metastasis, which accounts for most cancer-related deaths (Riggio et al., 2021; Dillekås et al., 2019). Recurrence, a form of progression occurring post-treatment, has significant physical and psychosocial impacts on survivors (Stewart et al., 2021). Consequently, individuals may experience fears related to the possibility of recurrence. This is to be expected, and some level of fear is thought to be of benefit to survivors as they will be alert to any indications that the cancer has returned. However, this fear can reach dysfunctional levels associated with a range of negative psychosocial outcomes (Simard et al., 2013). Accordingly, research has focused on fear of cancer recurrence (FCR) as a distinct psychological concern in its own right, and interventions to lower this fear to manageable levels are increasingly commonplace (Tauber et al., 2019).

While the seriousness of a recurrence diagnosis should not be understated, lifespan after the event varies according to a complex range of factors and though not always the case, is often considered to be a terminal diagnosis (Vogt et al., 2021). Even if so, a patient may subsequently live for several years and be monitored for signs of further progression which can signal the beginning of end-of-life care.

Indeed, fears related specifically to the progression of cancer are also a recognized psychological concern (Mehnert et al., 2006). Despite the possibility of experiencing further progression after a recurrence has occurred, the term fear of progression (FOP) is typically used interchangeably with FCR in the literature; this is evident in a widely utilized definition of FCR; “the fear, worry, or concern about cancer returning or progressing” (Lebel et al., 2016). Recent research (Coutts-Bain et al., 2022) has indicated that these are closely related but not identical constructs and should be treated separately in research and clinical practice (both before and after recurrence). Importantly, there is indication that fear levels are higher post-recurrence than pre-recurrence (Shim et al., 2010), yet FOP measurement remains underexplored at this time.

On the subject of measurement, FOP levels are often assessed (both pre and post recurrence (Mehnert et al., 2006; Shim et al., 2010)) using the 12-item Fear of Progression Questionnaire-Short Form (FOP-Q-SF) (Mehnert et al., 2006). But recent research has indicated the efficacy of the shorter Fear of Cancer Recurrence 4 item measure (FCR4) (Humphris et al., 2018) for FCR, and it would be of benefit to examine its use for FOP specifically.

Associations between elevated FOP levels and negative quality of life (QoL) outcomes have been established (Ban et al., 2021), indicating the need for a nuanced understanding of fear dynamics in this population after recurrence. With the indication that FCR and FOP should be treated as separate constructs it is pertinent to explore fear in patients specifically operationalized as FOP; this is possible since recurrence has already occurred, and fears will be unrelated to this event. This study aims to fill this gap in the literature by examining fear levels in patients post-cancer recurrence, an event that is known to have a significant impact on QoL (Stewart et al., 2021).

## 2 Methods

### 2.1 Research design

Using a sequential explanatory design over a period between June 2021 and December 2023, this mixed method prospective longitudinal study aimed to enhance the depth of understanding surrounding fear experiences post-recurrence. The quantitative phase involved administering three questionnaires, each a month apart to explore the trajectory of fear levels and associated factors. This longitudinal design is particularly relevant given indications that FCR levels may exhibit fluctuations over time (Deuning-Smit et al., 2022). Indeed, longitudinal analysis has not previously been conducted when fear is operationalized as FOP post-recurrence, and so establishing trajectories in this population for the first time will be of use to future research. The qualitative phase encompassed individual semi-structured interviews which took place within a month of final questionnaire data collection. This mixed methods approach enabled a more comprehensive exploration of fear dynamics than achievable through a single methodology, enhancing the robustness of the study’s findings (Dawadi et al., 2021).

### 2.2 Questionnaire

A questionnaire was developed for the purposes of the study comprised of both validated and unvalidated scales, and this was administered at baseline, and one- and two-months following baseline, respectively. The following components were included:

The *12-item Fear of Progression Questionnaire-Short Form (FOP-Q-SF)* (Mehnert et al., 2006). On a five-point scale, participants are asked how often a particular symptom of FOP is experienced from 1 (never) to 5 (very often).

The *Fear of Progression 4 item measure (FOP4)* is identical to the Fear of Cancer Recurrence 4 item measure (FCR4) (Humphris et al., 2018), but with references to cancer recurrence changed to cancer progression. This scale was included alongside the FOP-Q-SF to check for convergent validity.

*The EuroQol 5-dimensional questionnaire five-level version (EQ-5D-5L)* (Herdman et al., 2011) was used to measure health-related quality of life in participants. One of five ratings is given to each category: no problems, slight problems, moderate problems, severe problems, or extreme problems. A number can be applied to each rating (e.g., a ‘one’ would indicate no problems and ‘five’ would indicate extreme problems), and the five are combined (such as 11,232) to express the health of the patient.

*The Hospital Anxiety and Depression Scale (HADS)* (Zigmond and Snaith, 1983) was included in order to measure anxiety and depressive symptoms, as well as test for convergent validity with the FOP measures. This is a 14-item scale with seven items measuring anxiety and seven items measuring depression, which are scored separately.

*The Clinical Care COVID-19 Anxiety Scale (CCAS)-* As the research was conducted during the COVID-19 pandemic, four items designed to measure fears about COVID-19 and its effect on cancer and treatment were included. This a 5-point scale that asks patients to indicate how often they worry about certain aspects of the pandemic on their illness and treatment from ‘not at all’ to ‘all the time’ (Yuan et al., 2023).

### 2.3 Qualitative interview schedule

An interview schedule was developed prior to the commencement of the study and featured 10 questions and additional prompts based on questions in the questionnaire.

### 2.4 Study population

The sample required patients with a recurrence of their cancer. The following inclusion criteria were applied:

- Previous treatment of an initial cancer diagnosis.
- A confirmed diagnosis of cancer recurrence within 3 years.
- Able to understand English.

Participants were patients from an NHS specialist cancer treatment trust with sites in London and Surrey, UK. Ethical approvals were obtained from the University of Stirling NHS, Invasive or Clinical Research Panel and the NHS London – Stanmore Research Ethics Committee.

The cancer care teams identified and approached patients eligible for the study. Participants completed the questionnaires over a phone call or were posted a pack of three questionnaires, after completion of the first questionnaire the participants were contacted twice more monthly to repeat the questionnaire. Participants selected for interview were contacted after they had completed this phase, with this sub-sample based on a sampling matrix to cover a range of clinical and demographic factors.

### 2.5 Data analysis

#### 2.5.1 Quantitative data analysis

Quantitative data analysis was conducted with Statistical Package of Social Sciences (SPSS) version 28. Any missing values were imputed in Missing Completely at Random (MCAR) principles. Descriptive statistics of questionnaire data were reported, and inferential statistics were calculated via multiple regression analysis to predict FOP scores from cancer type, gender, age, and time since recurrence. As a longitudinal study, an overall average was taken from mean scores at each data collection time point for this purpose. For the longitudinal data, repeated measures ANOVA were conducted to test for an effect of time on each of the questionnaire components. Pearson Correlation Coefficients were calculated to test for convergent validity between the FOP questionnaire components and the HADS subscales. Cronbach’s Alpha was measured to test for the internal consistency of the CCAS (as a scale in development), and the FOP4 (as it has not been operationalized in this form).

#### 2.5.2 Qualitative data analysis

Qualitative data was analyzed using the six step inductive thematic analysis technique from Braun and Clarke (2006) using QSR NVivo 20. Two interview transcripts were coded independently. After reaching consensus one researcher coded the remaining transcripts for analysis. Themes were developed in the same manner from these codes and all themes were reviewed by the research team to validate the results. Continuous checking for data saturation was conducted by two members of the research team- represented by fewer and fewer themes emerging.

#### 2.5.3 Integration of data sets

Integration firstly took place firstly at the study design level (Fetters et al., 2013). Integration was also carried out at the methods level; through the concept of *building*; where results from one data collection procedure informed the next (Creswell and Clark, 2017) (via the sampling matrix). Lastly, integration took place at the interpretation and reporting level by *integrating through narrative*, in which both components are described, using a contiguous approach, as well as through a *joint display*, where data are brought together visually (Fetters et al., 2013).

## 3 Results

In the first phase of the research 44 patients were approached, of which 34 (77%) agreed to take part. Twenty-nine completed all three questionnaires, with five completing either one or two. Descriptive and clinical statistics of participants are displayed in Table 1.

**Table 1:** Demographic and clinical variables of participants.

Variable	*N*
Phase 1	
Female	19
Male	15
Mean age (SD)	64.26 (11.94)
Cancer type	
Breast	17
Prostate	9
Bladder	2
Rectal	2
Renal	1
Melanoma	1
Leiomyosarcoma	1
Non-Hodgkin’s	1
Mean months since initial diagnosis (SD)	68.78 (71.45)
Mean months since initial recurrence (SD)	42.32 (50.65)
Phase 2	
Female	6
Male	4
Mean age (SD)	68.2 (13.67)
Cancer type	
Prostate	4
Breast	3
Bladder	2
Melanoma	1
Mean months since recurrence (SD)	55.2 (67.49)
FOP score	
Low-Moderate	7
High	3

### 3.1 Questionnaire scoring

A repeated measures ANOVA was conducted to test for an effect of time on each of the questionnaire components and no significant effect was found, indicating that FOP, anxiety, depression, and COVID-related fears remained stable throughout the duration of the study. See Table 2 for questionnaire scoring across timepoints alongside longitudinal analysis.

**Table 2:** Average questionnaire scores and longitudinal analysis of questionnaire component scales.

	Baseline		Follow-up 1		Follow-up 2		df	F	p	Partial eta squared
Measure	M	SD	M	SD	M	SD				
FOP-Q-SF	31.32	9.42	31.50	9.30	31.12	8.60	2, 32	0.063	0.939	0.002
FOP4	12.26	4.05	12.71	4.33	12.52	3.80	2, 32	0.406	0.668	0.012
HAD-D	3.93	2.49	3.95	3.09	4.06	2.90	2, 32	0.110	0.896	0.003
HAD-A	5.77	3.05	5.42	2.89	5.90	3.13	2, 32	0.945	0.394	0.028
CCAS	7.53	3.86	7.89	3.81	7.34	3.38	2, 32	0.882	0.442	0.024
Measure	Baseline (mode)	Follow-up 1	Follow-up 2	
EQ-5D-5L	21221		21221		21221	

### 3.2 Fear of progression

With a score of 34 in the FOP-Q-SF indicating dysfunctional levels of fear, the mean score peaked at 31.5. On average, 35.3% of participants registered a score 34 or over (though this peaked at 38.2% at one measurement point). For the smaller FOP4 scale, high scores were considered 14 and over. Mean scores peaked at 12.7, which also indicated moderate but not clinically dysfunctional levels of fear. Consideration is given to the correlation between these measures below.

Multiple regression analysis suggested that gender, age, and time since recurrence did not statistically significantly predict FOP, *F* (4, 29) = 2.74, *p* = 0.48, adjusted R^2^ = 0.174. These findings are summarized in Table 3.

**Table 3:** Multiple regression analysis of demographic and clinical variables on fear of progression.

Predictor variable	B	Std. Error	Beta	t	p	95% CI	
						Lower bound	Upper bound
Time since recurrence	−0.024	0.028	−0.147	−0.859	0.397	−0.083	0.034
Age	0.42	0.144	0.059	0.289	0.774	−0.253	0.337
Gender	−10.388	3.943	−0.615	−2.635	0.13	−18.452	−2.324
Cancer Type	2.070	0.947	0.440	2.187	0.037	0.134	4.007
Constant	40.194	7.401	-	5.431	<0.001	25.057	55.330

To assess the relationship between health-related QoL and FOP, mode health status scores for each dimension of the EQ-5D-5L were compared to mean FOP-Q-SF scores using Spearman’s rank correlations. No significant correlations were identified between the dimensions and FOP scores, except for pain; suggesting that pain is positively associated with increased FOP.

### 3.3 COVID-19 concerns

For the CCAS, any scores that exceeded two standard deviations above the mean were considered high levels of fear. If taking the average of means as the guideline, this would require participants to score 14 and over, indicating that fears related to cancer and COVID-19 were generally low.

Multiple regression analysis suggested that and none of the demographic and clinical variables collected predicted CCAS scores, *F* (3, 30) = 1.632, *p* = 0.193, R^2^ = 0.184.

### 3.4 Reliability and validity

Analyses suggested that the FOP4 and CCAS showed high levels of internal consistency (Cronbach’s Alpha = 0.95 and Cronbach’s Alpha = 0.93, respectively). These results surpass the traditionally cited level of reliability necessary for new scales (Taber, 2018). The FOP4 also showed high convergent validity to the FOP-Q-SF and the HADS anxiety and depression sub-scales (see Table 4).

**Table 4:** Convergent validity of fear of progression measures.

	Pearson correlation coefficients			
	FOP4	FOP-Q-SF	HAD-D	HAD-A
FOP4	1	0.697*	0.474*	0.556*
FOP-Q-SF	0.697*	1	0.441*	0.599*
HAD-D	0.474*	0.441*	1	0.700*
HAD-A	0.556*	0.599*	0.700*	1

^*^Correlation is significant at the 0.01 level (2-tailed).

### 3.5 Qualitative results

Ten participants were recruited for the qualitative phase via telephone no more than a month after they completed their final questionnaire. This was based on a sampling matrix that sought to capture a range of fear scores, ages, cancer types, and times since recurrence. Thematic analysis resulted in the development of 4 key themes, which are discussed below with example quotes.

#### 3.5.1 From recurrence to progression

Participants acknowledged the incurable nature of their cancer diagnosis and the need for ongoing monitoring for disease progression. This theme captures their experiences with initial diagnosis, recurrence, and anticipated progression. Many described the recurrence as a period of shock and frustration, especially after initial successful treatment.

*“After a year or so, tests showed it had come back and metastasised, which was a terrible shock; actually, rather frustrating, I wasn’t upset as such.” —Bl1 (83 years old, bladder cancer patient).*

Conversely, others described their feelings at this time as less of a shock but acknowledged that the diagnosis was now incurable.

*“But with the recurrence it was less of a shock but more of an acceptance. A sad one, but an acceptance. There were treatment options, so it wasn’t total doom and gloom, but I had to accept it was never going to be gone like before.” — B2 (72 years old, breast cancer patient).*

This acceptance was echoed by several participants who expressed being at peace with their current condition and that further progression will occur.

*“I know I’m near the end so I suppose I want to maintain a certain quality of life at this point, if it was to go downhill soon then I would rather it did not take too long. And that’s not me being morbid or anything I’m quite relaxed really.” — Bl1 (83 years old, bladder cancer patient).*

Some participants felt that they were living with a great deal of uncertainty about further progression, so found planning difficult.

*“But the other way I find it exceedingly challenging is decision making. I find it very, very difficult to know, like when I was initially diagnosed, they thought I’d be dead very quickly and so I gave up work in order to spend time with my kids and changed my life.” — B3 (55 years old, breast cancer patient).*

Despite living in a state of relative acceptance, several participants reported feeling anxious in the period before a scan appointment or before receiving subsequent results.

*“The only time I worry is probably a couple of days leading up to the scans because the scans will show whether the cancer is expanding, or whether it’s contracting, or whether it’s stabilised. Because, dependent on the result of that, you know, I may or may not stay on this trial.” — P4 (80 years old, prostate cancer patient).*

Other participants worried about progression of their cancer affecting their options for treatment.

*“I try not to think about it. I know by now I will not be cured but as long as I can keep going with treatments, and you know it does not progress so much that it’s not possible, I’ll just about manage.” — B1 (40 years old, bladder cancer patient).*

#### 3.5.2 Experience across lifespan

This theme underscored the variability in cancer experiences influenced by life events and personal circumstances, such as age, parenthood, or prior cancer history. Several participants were of retirement age; however, a younger breast cancer patient reported challenges not only in maintaining physical capacity for work but also in planning and managing substantial workloads.

*“… do I go back to work, do I not go back to work, and if I go back to work do I live the same lifestyle I had before? You know, I used to be manic and do loads of things simultaneously and so, do I want to take that on if I’m going to be dead? What’s the point? — B3 (55 years old, breast cancer patient).*

The same participant expressed worries about her ability to perform her usual activities for her family, as well as the implications of the diagnosis on her husband and children.

*“I need a lot of practical support for two young children that needed picking up from school and I was in hospital for hours” … but also, it’s very difficult for him (her husband) to come to terms with what’s happened to me and I think possibly my children… because they are just, you know, helpless observers.” — B3 (55 years old, breast cancer patient).*

Older participants commonly expressed minimal concern regarding their children, primarily because they were already adults and more independent.

*“Of course, you would worry about your family but they are not young anymore. If you’d asked me that 20–30 years ago my answer would have been different.” –M1 (76 years old, melanoma patient).*

Similarly, several older participants expressed sympathy for younger patients with cancer, despite their own diagnosis.

*“I’m in my 70s, I’ve had a great life- not that I want it to end anytime soon, but someone younger, especially with the recurrence, that must be hard. Again, I’m by no means saying it’s easy for me, but I bet 20 years ago I would be much more distressed.” — P3 (76 years old, prostate cancer patient).*

Some participants indicated that their previous experience with cancer had mitigated some of their concerns regarding their current condition.

*“Cancer has been around me for a long time and with this more recent recurrence it’s not a joyous position to be in, but I’m somewhat used to it and in all honesty. I just put it behind me.” — Bl2 (78 years old, bladder cancer patient).*

#### 3.5.3 Managing the impact

This theme was developed to capture the sources of comfort and support that participants identified as helpful in coping with their condition. These sources included people, places, activities, and personal thought patterns. Related to their acceptance of their condition and the possibility of future progression, some participants indicated that they had learned to manage adverse life events, demonstrating a self-perceived resilience.

*“A few weeks ago, I started on a new treatment and that’s basically the current state of play. In terms of how I’ve dealt with that, as well as the rest of it, you know, pretty well. But I think I’ve always been quite good at that, when things get rough.” — M1 (76 years old, melanoma patient).*

Some participants stated that taking part in activities took their mind of their cancer and helped them to cope with the condition.

*“Well, while you are exercising, you do take your mind off things, but there are proven benefits of exercise in terms of, you know, dealing with the cancer, dealing with the side effects of chemotherapy… I said okay, well, how can I help myself?… there’s three key areas which are exercise, nutrition, and mental health, which I could control myself. — P1 (58 years old, prostate cancer patient).*

Participants often emphasized the importance of social support from their friends and family in helping them cope with their condition.

*“In this country I have a wide range of friends no family of course and thanks to the miracle of video calls I speak to my wife and granddaughter who are abroad every night without fail.” — M1 (76 years old, melanoma patient).*

Further, some felt that relations were enhanced with relatives after their diagnosis.

*“I’ve got a great family who are always there for me. Since the diagnosis, they have really rallied around me, and I know if I need anything they will be there.” — P1 (58 years old, prostate cancer patient).*

Though some participants stated that they did not want to feel like they were burdening their loved ones.

*“I certainly do not discuss it very often with my wife and she knows as much as I do, and I do not want to make her unhappy by talking about my health.” — Bl2 (78 years old, bladder cancer patient).*

Several participants found comfort in encountering others who were also navigating their journey with cancer.

*“I can recall chatting to others whilst waiting for appointments, it can be a bit of a reminder that others are going through it. A bit of camaraderie I suppose.” — Bl2 (78 years old, bladder cancer patient).*

The interactions with healthcare professionals throughout the cancer journey were frequently discussed. Some emphasized the significance of developing positive relationships with healthcare staff.

*“The staff have been great; they really care about me. They call in case I have any side effects or if I’m not feeling well. If I call them and leave a message, they’ll call me back within an hour. They’ve been very, very, very cooperative here.” — B1 (40 years old, breast cancer patient).*

Others felt that they had had negative experiences with staff due to poorly perceived communication.

*“But basically, he stood there looking traumatised himself clutching an MRI scan and the doctor told my husband that his wife’s going to die, which was very, very blunt. It’s like delivering news with a mallet — B3 (55 years old, breast cancer patient).*

In addition to discussing communication with staff, participants expressed a strong desire to actively participate in their treatment decisions and to possess a comprehensive understanding of their medical condition.

*“I’m never shown anything that I can understand. Maybe they do not want to alarm me, maybe they do not have time to bring scans down to consultations. I’m never truly in the picture.” — Bl2 (78 years old, bladder cancer patient).*

The interviews also revealed the positive impact of receiving treatment at a specialized cancer hospital.

*“I said to her (the doctor), ‘is it the right thing we are doing coming to see you?’. And she said, well… ‘you know you are at the centre of excellence in cancer research’… she had managed to relatively stabilise things and I’ve had high quality life from that time, it’s been great being here really.” — P4 (80 years old, prostate cancer patient).*

#### 3.5.4 Cancer and COVID-19

The final theme was developed to underscore participants’ discussions concerning the COVID-19 pandemic’s implications for their cancer treatment and broader lives. A commonly expressed concern from participants revolved around the potential impact of the pandemic on their ongoing cancer treatment.

*“And there was that initial worry but the staff at the hospital reassured me that treatments would be ongoing, even now the worry is more of inconvenience, if I was to miss an appointment because I had covid, rather than, you know, worrying about physical issues from catching it.” — B2 (57 years old, breast cancer patient).*

Participants conveyed gratitude for receiving treatment at a specialized NHS hospital dedicated to cancer care, which remained largely unaffected by treatment interruptions stemming from the COVID-19 pandemic and was not utilized for COVID-19 patients.

*“I think the difference is it’s a specialist hospital, is not it? They are not overrun with lots of other things. So, during COVID, you know, you felt quite privileged to be going to there….” — P4 (80 years old, prostate cancer patient).*

Several participants noted that their greatest fear of contracting COVID-19 occurred at the outset of the pandemic. However, by the time of the interviews, they expressed feeling safer due to receiving vaccinations.

*“But you know we have had all of our vaccines so it’s easier now, but not to say I’m throwing caution to the wind, but they give you that confidence you know? So, no I’m not worried anymore.” — B2 (57 years old, breast cancer patient).*

But several felt that they still had to take extra precautions.

*“I know you do not have to, but I just do not want to throw caution to the wind. I’m concerned about it when, like right now the weather is warm, it will not spread as much, but once it gets colder, the colder months, then it spreads. So, I try to minimise my outings to a very basic level, these days it’s minimal, really just the hospital.” — B1 (40 years old, breast cancer patient).*

Some participants felt that, because they were taking more precautions than people not at high risk of health complications, they were looked on negatively.

*“When it when we first put into lockdown, everyone was in it together. But for people who are now vulnerable, you feel a bit forgotten. This is probably wrong of me, but I feel resentful of people who do not have any issues, who show no consideration for people who may have to be more careful. You feel a bit judged by them too.” — P2 (64 years old, prostate cancer patient).*

#### 3.5.5 Integration of findings

In addition to integrating through narrative, a joint display (Fetters et al., 2013) in the form of an integrated visual display (Table 5) displaying quantitative and qualitative findings next to each other is included (McCrudden et al., 2021). As can be seen, the integration of results was judged to provide expansion to each of the main topics.

**Table 5:** Integration of quantitative and qualitative findings.

Subject	Quantitative results	Qualitative results	Integration
Fear of cancer progression	Moderate FOP was present in the sample, and over a third of participants reached dysfunctional levels. Top three highest scoring questions on the FOP-Q-SF were ‘worrying what will become of family if something happens to me’, ‘being afraid of disease progression’; and ‘being nervous prior to doctor’s appointments or periodic examinations’. Three lowest scoring questions were ‘being afraid of not being able to work anymore’, ‘being afraid of pain’ and ‘being afraid of relying on strangers for activities of daily living’.	*“I know by now I will not be cured but as long as I can keep going with treatments, and you know it does not progress so much that it’s not possible I’ll just about manage.” — B1 (breast cancer patient).* *“Yes of course, it is something I think about, particularly nearing scan appointments and whatnot, I’ve just got to hope that it’s kept at bay for a while, but it does enter my mind and I might be a bit tetchy close to the time.” — B2 (breast cancer patient).* *“I certainly do not discuss it very often with my wife and she knows as much as I do, and I do not want to make her unhappy by talking about my health.” — Bl2 (bladder cancer patient).*	Expansion Qualitative and quantitative findings converge on a general level with FOP expressed throughout both phases. A degree of convergence was also found when comparing individual questions from the questionnaire to the interview transcripts. Expansion resulted from further discussion that was possible from the interviews. For example, more nuanced answers were given in regard to the timing of fear levels.
Psychological concerns	On average, participants scores peaked at 4.06 on the depression subscale of the HAD throughout the study, which falls within the ‘normal’ score of 0–7. For the anxiety subscale mean scores peaked at 5.90, within the ‘normal’ score of 0–7.	*“The only time I worry is probably a couple of days leading up to the scans.” — P4 (prostate cancer patient).* *“No in all honesty I keep things to the back of my mind and do not worry about it I can still do most things I like and if I cannot then there’s no point worrying about it so equally there is no point in anticipating it.” — M1 (melanoma patient).* *“And so, when something happens to you and you think to yourself, this is not good. You then have to reset your focus and saying, well, I’m here now. So what do I do about it? To get through it?.” — P2 (prostate cancer patient).*	Expansion Both components of the study complement each other in the sense that when talking about day-to-day life many participants were not very anxious or depressed. Again, qualitative findings allowed for more discussion on how these levels may vary, e.g., giving more insight into coping methods.
Health-related quality of life	On average, mobility scores, usual activities, and pain/discomfort were rated as 2, indicating slight problems. Self-care and anxiety and depression were rated as 1 throughout, suggesting no problems, on average.	*“I did like to go walking with my friends and just kind of socialise, but truth be told I do not have the energy these days with the treatment, that’s a little frustrating, I did like that.” — Bl1 (bladder cancer patient).* *“The previous time I was on chemotherapy I was able to run and cycle and I was really, really quite active. But this time because I’ve got tumors around the base of my skull, they recommended that I did not do anything too active.” — P1 (prostate cancer patient).* *“At the moment it’s been, a little down because of a new growth that’s affected my stability and making me quite dizzy.” — B1 (breast cancer patient).*	Expansion Both sets of findings support each other; not every participant expressed issues in their health-related QoL, but others did, and primarily in relation to their comfort and ability to perform usual activities. Interviews allowed participants to go into more detail than was possible with just the questionnaire, providing more comprehensive answers and greater insight.
COVID-19 pandemic	Mean scores on the CCAS peaked at 7.89 out of a possible 20, indicating that fears related to cancer and COVID-19 were generally low.	*“I suppose it did worry me at the start. I’m more concerned now about missing my appointments if I were to catch it again, that would be a real pain. But in terms of the illness, no not so much anymore.” — P3 (prostate cancer patient).* *“But you know we have had all of our vaccines so it’s easier now, but not to say I’m throwing caution to the wind, but they give you that confidence you know? So, no I’m not worried anymore.” — B2 (breast cancer patient).* *“You know, I was. I’m just now, if I catch it, that’s not great. But I’m not like so worried about catching it because I have not caught it in the two years, and I’ve gotten my shot, and my booster. So no, I’m not really worried.” — B1 (breast cancer patient).*	Expansion Quantitative findings suggest a generally low level of fear. This is supported by the qualitative data, which indicates participants were not unduly worried about the pandemic at the time of interviewing. However once again, qualitative data illuminated responses in a more detailed manner (e.g., much of the concern about having COVID-19 was in relation to missing a subsequent hospital appointment).

## 4 Discussion

### 4.1 Fear of progression

The findings in this project indicate that on average, patients with recurrent cancer have low-moderate FOP, but over a third displayed dysfunctional levels. Integrated findings from the mixed methods approach suggest that some consequences of cancer progression concern participants more than others, and it is possible to identify these. FOP is not usually measured in recurrent cancer groups, but in non-recurrent cancer the number reaching dysfunctional levels varies (Niu et al., 2019; Hinz et al., 2015), and so current findings fall within the range previously reported in different populations. Increased anxiety, depression and pain were associated with higher levels of FOP, in line with the literature (Crist and Grunfeld, 2013). Though it was expected that other aspects of health-related QoL would be associated with higher FOP (Simard et al., 2013) and this was not the case- this is worthy of investigation in larger samples.

A novel finding is that levels of FOP remained stable over the course of three months. This is a first step in the understanding of the trajectory of FOP after recurrence and suggests that if a patient scores highly for FOP, then they are likely to continue to score highly. Though subject to individual fluctuations, evidence suggests that patients with a primary diagnosis of cancer also have fairly stable levels of FCR/FOP up to ten years after baseline assessment (Butow et al., 2021; Götze et al., 2019). For the current research this stability may be due to a range of factors- it is plausible that there is a degree of acceptance in some regarding further progression after recurrence, which is evident in the qualitative phase. This could explain fear not increasing for some. For those who scored highly, evidence suggests that individual psychological characteristics are more consistent predictors of fear trajectories than sociodemographic and disease-related factors in general cancer populations (Sun et al., 2024). This may also be the case for FOP post-recurrence; however, longer term research is needed to assess if FOP trajectories will continue to remain stable as FCR does.

Interestingly, several participants expressed acceptance at receiving an incurable diagnosis but with a desire to continue treatments that would increase their lifespan and allow them to maintain acceptable QoL. This suggests that many of the participants wished to avoid further progression which a medical appointment may reveal- this appears to be an important source of FOP at this time.

### 4.2 Determinants of measured outcomes

The demographic and clinical variables collected did not predict FOP levels. There are often contradictory findings about the role of clinical variables in relation to fear levels (Simard et al., 2013), but higher FCR/FOP levels are often linked to those of lower age, and those who have young children (Simard et al., 2013; Crist and Grunfeld, 2013). Tentative support for this is gleamed from the qualitative phase of the current research, where those with young children expressed concerns about their caregiving commitments and the emotional toll placed on a young family. Relatedly, previous research (Fugmann et al., 2023) suggests that parents with cancer face a multitude of issues related to caregiving and these problems are not adequately addressed in current patient care protocols.

Several participants described adaptive coping strategies, and evidence suggests that these are linked to positive QoL and low levels of distress in cancer patients (Seiler and Jenewein, 2019). Social support was highlighted by several participants as an important factor in their ongoing well-being. Crucially, types of social support most helpful for patients with cancer differ based on their unique circumstances. For instance, emotional support might be perceived as overly intense or distressing for the patient; instrumental support could be seen as unwanted if the assistance comes from someone with whom the patient lacked a close pre-diagnosis relationship; and informational support may be unhelpful if perceived as vague or inadequate (Wanzer and Czapla, 2022). Research involving general cancer patients suggests that greater perceived social support is linked to lower FCR, with no significant differences between types of social support offered (Lu et al., 2023). It would be of interest to explore if types of social support are valued more than others by patients in the time after a recurrence.

## 5 Limitations

Though the sample size for both phases is within the range of similar previous research (Stewart et al., 2021; Almeida et al., 2019), this study did nonetheless feature a relatively small sample size and so caution should be taken when interpretating results (Lakens, 2022). As noted, not every participant completed questionnaires at all three data collection points; these participants did not respond to follow-up requests from the research team. The reasons for withdrawing were not captured and the research team did not have access to full patient records so no distinction could be drawn between the characteristics of those who left and those who remained in the study. As such there is a risk of attrition bias in the study results (Beller et al., 2022).

It is crucial to repeat at this point that the current research took place within a cancer specialist hospital site, affecting generalizability. Previous research suggests an association between patient satisfaction and cancer treatment outcomes in healthcare facilities with a higher number of specialist staff (Ganti et al., 2017; Griffiths et al., 2013; Vernooij et al., 2009). Additionally, the negative impact of COVID-19 suppression measures on cancer treatment has been well-documented (Teglia et al., 2022); as the current study was conducted at a site where cancer treatment was relatively unaffected by the pandemic, this may mean results would differ at a non-specialist site during the pandemic.

## 6 Research implications

To the best knowledge of the researchers this is the first longitudinal study examining FOP in patients who have experienced a recurrence of their cancer. Similar designs in future research would greatly enhance findings from this study (Dinkel and Herschbach, 2017). This is also thought to be the first study featuring a qualitative examination of FOP in this population, and there is a recognized need to increase such research related to FCR/FOP (Almeida et al., 2022).

Results indicate that the FOP4 is a useful measure in this nature of research and may be suitable for exclusive use in future research with similar patients. Being of a smaller size means that the time required of participants, who may be experiencing treatment side effects and other physical issues cause by their cancer, is lessened.

### 6.1 Contributions toward fear of progression theory

Of the theoretical models used to understand FCR/FOP, the most exhaustive and evidence-based is thought to be Leventhal’s self-regulation model of illness (Leventhal et al., 2012), also known as the Common Sense Model of Illness (CSM). A version of this model specifically adapted for FCR by Lee-Jones et al. (1997) posits that levels of fear depend on the cognitive reaction to illness. This model suggests that internal stimuli (e.g., cancer symptoms) are interpreted by the individual as a sign that the cancer has possibly recurred or progressed. Simultaneously, external stimuli (e.g., medical appointments) will increase worry about recurrence or progression. Subsequently, this subjective perception and accompanying emotions (such as fear) lead to appraisal of their condition and relevant coping strategies.

The constructs of the adapted CSM model were partially supported by the thematic analysis of the qualitative phase of the research. External cues (particularly contact with healthcare staff and an individual’s predisposition and past coping style) appear to have led to participants considering their risk of cancer progression and produce a relevant emotional response and certain behavioral responses (e.g., limited planning for the future), and psychological responses were evident. It should be noted, from interview transcripts, that internal cues (such as pain and tiredness) were present, but these did not appear to be acting as a cue to begin worrying about the possibility of cancer progression.

Constructs from the CSM may account for variation in FOP levels in patients with cancer recurrence, though more direct assessment of these would be beneficial in future; it is plausible that the model should be adjusted to explain the mechanisms of FOP as a distinct concept, with differing predictors of FCR to FOP now expected (Coutts-Bain et al., 2022). Current results further lend credence to the notion of differentiating between these concepts; the need to clarify whether one is referring to FCR or FOP adds complexity when examining patients after a recurrence has occurred.

### 6.2 Fear of progression intervention development

It is recommended that psychosocial interventions should be designed for patients with recurrent cancer, in line with FCR research (Tauber et al., 2019). FCR/FOP terminology should be carefully considered when selecting a theoretical model to inform interventions: interventions solely tailored for FOP may have lesser efficacy compared to those for FCR, potentially owing to distinct underlying mechanisms (Coutts-Bain et al., 2022). Current findings suggests that FOP is reasonably stable after recurrence, in line with FCR/FOP generally (Simonelli et al., 2017); however, participants indicated that fear levels in relation to further cancer progression rise around the time of medical scans or appointments; this is worthy of consideration.

## 7 Clinical implications

In patients with incurable disease it is suggested that a good professional relationship and communication levels with healthcare staff have positive consequences for mental well-being (Chen et al., 2023). In the current study, participants expressed the importance of their relationships with healthcare staff. Interestingly, there is evidence to suggest that patients with advanced cancers are often less satisfied with healthcare professionals (Alessy et al., 2022), and dissatisfaction with healthcare staff has been linked to higher levels of FCR/FOP before recurrence occurs (Anderson et al., 2021). With a multitude of competing priorities for healthcare staff the principles of person-centered care may not always be actioned satisfactorily (Ocloo et al., 2021). Moreover, research indicates that oncology specialists often perceive their primary role in patient care as focused on monitoring for recurrence and managing symptoms, while viewing psychosocial well-being as within the purview of primary care providers (Lisy et al., 2021). To address such challenges, policymakers should focus on diligent implementation of strategic plans, such as the NHS Long Term Plan (Alderwick and Dixon, 2019).

In the current research COVID-19 related concerns were generally found to be low in relation to cancer and its care. Certain aspects, particularly related to canceled appointments, were a source of worry for participants at interview. Previous research has indicated that the pandemic created delays along treatment pathways for cancers and has been a source of psychosocial concern for both those with cancers and with other benign conditions requiring ongoing hospital care (Barone et al., 2022; Crocetto et al., 2022; Ferro et al., 2021). As such, careful consideration should be given to any impact on treatment, and to the effect this may have on patients’ wellbeing.

With the incidence of cancer in the UK expected to rise and NHS cancer services facing challenges in recovering from the impact of the COVID-19 pandemic (Aggarwal et al., 2023), the complexities of cancer care are exacerbated. Notably, more adults are living with treatable but incurable cancers (White et al., 2021) and psychosocial concerns persist throughout the cancer experience. Findings from this research support the idea that the emergence of FOP should be monitored as part of ongoing care.

## Data Availability

The raw data supporting the conclusions of this article will be made available by the authors, without undue reservation.
